# The Effect of the Educational Program on Iranian Premature Infants’ Parental Stress in a Neonatal Intensive Care Unit: A Double-Blind Randomized Controlled Trial

**Published:** 2014-10

**Authors:** Noushin Beheshtipour, Seyedeh Marzieh Baharlu, Sedigheh Montaseri, Seyed Mostajab Razavinezhad Ardakani

**Affiliations:** 1 Community Based Psychiatric Care Research Center, Department of Pediatric Nursing, School of Nursing and Midwifery, Shiraz University of Medical Science, Shiraz, Iran;; 2 Student Research Committee, School of Nursing and Midwifery, Shiraz University of Medical Science, Shiraz, Iran;; 3 Department of Pediatric Nursing, School of Nursing and Midwifery, Shiraz University of Medical Science, Shiraz, Iran;; 4 Department of Pediatrics, Neonatal Research Center, Shiraz University of Medical Science, Shiraz, Iran

**Keywords:** Education, Intensive Care Units, Parents, Premature Infant, Stress

## Abstract

**Background: **Hospitalization in neonatal intensive care unit (NICU) leads to a lot of stress and shock to the parents. Nurses, as the primary sources of information, could play an important role in reducing their stress. The aim of this study was to determine the effect of educational program on the premature infants’ parental stress in NICU.

**Methods: **This double-blind randomized controlled trial study with a pre-and post-test and follow up design was conducted from February 2013 to March 2014. Sixty parents in Hazrat Zainab hospital affiliated to Shiraz University of Medical Sciences were randomly allocated into the intervention (received educational program) and control groups (received routine care). The valid and reliable ”Parental Stressor Scale: Neonatal Intensive Care Unit (PSS: NICU)“ was used to measure the parental stress. In the intervention group, information about general condition of the baby, the equipment and unit’s environment, spouse support, and problem solving strategies were given. The data were collected the second day after admission, fifth day after admission, and a week after the intervention. The data were analyzed in SPSS (Version 14), using t-test and repeated measures analysis of variance.

**Results: **In the second day after admission, the mean score of stress in premature infants’ mothers and fathers in the intervention group were 94.79±14.28 and 76.77±16.39, respectively. In the control group, it was 94.48±20.03 and 92.30±21.95 for mothers and fathers. After the intervention in the fifth day of admission, a significant difference was observed between the two groups concerning the premature infants’ maternal (t=-5. 23, P<0.0001) and paternal (t=-6.17, P<0.0001) stress. Moreover, a week after the intervention, the stress mean scores were (in the intervention group: for mothers=59.72±13.55 and for fathers=61.22±18.00), and (in the control group: for mothers=86.75±12.12 and fathers=84.70±18.46). Moreover, a significant difference was shown between the two groups concerning the premature infants’ maternal (F=23.05, P<0.0001) and paternal (F=17.35, P<0.0001) stress mean scores during the three study periods.

**Conclusion: **The results of this study showed that parents’ educational program can reduce their stress and they can spend their energy to support and care for their baby instead of coping with stress.

**Trial Registration Number: **IRCT2013121515810N1

## Introduction

Pregnancy, childbirth and the postpartum period is a milestone in one’s life; this experience deeply affects the families and has long-term and important effects on the society.^1^ About 13 million births annually are premature which is the main challenge in perinatal care and in 2008, it had a prevalence of 12.3%.^2^ In Iran, a cross-sectional study conducted in Yasuj, estimated the incidence of preterm birth of infants to be 4.8% and low-birth-weight 7.6%.^3^ In fact, prematurity and LBW are two of the main causes of infant mortality^4^ and infant mortality rate was reported 3.18 in 1000 births in Iran.^5^ Infants born before 37 weeks from the first day of the last menstrual period are called premature.^6^ These babies are very delicate, and, physically, many of their body organs are still not fully mature.^7^ Parents who have waited months to have a healthy, beautiful and perfect baby are shocked with the birth of the baby that needs to stay in the fully equipped and different neonatal intensive care unit, sot hey suffer from a lot of stress because of the disruption and loss of parental control and separation from their baby.^8^^-^^12^ In addition, the appearance and behavior of the infant, and the unit’s environment like light, sound and unfamiliar equipment, tubes and devices which are connected to the baby cause their stress to be doubled.^12^^-^^15^ Parental stress is higher especially during the first week of hospitalization of infants.^16^ In Iran, premature infant with anomalies is viewed as a family flaw and having strong and healthy infant is so important and great for them because the parents of an NICU infant are shameful of social stigma.^17^ Moreover, impairment of breast feeding in the mother with acute physical and mental stress has been reported in experimental studies. This happening disturbs milk ejection reflex because of reducing the release of oxytocin during breast feed. It could reduce milk production by incomplete breast emptying at each feed. In another study, it was shown that both maternal and infant stress during each period of labor and delivery resulted in delayed onset.^9^ From mental aspect, the mothers of premature infants, themselves, are exposed to stress^9^ because they are more in touch with their newborn babies and they are directly involved in the treatment done for the baby. Since the fathers are responsible to pay for treatment, social and economic status and poor performance of the family influence their anxiety and depression, especially fathers, so fathers also need emotional support.^11^ However, mothers and fathers differ in terms of their concerns,^8^ but it depends on the extent of their relationship with their baby and their perceptions of their parental role.^15^ Establishing a support system for parents of NICU infants seems urgently needed to improve parent-infant bonding.^17^ Since during hospitalization of the infants, the Neonatal Intensive Care Unit (NICU) staff, especially nurses, are in connection with parents more than any other person and on the other hand they are more aware of the baby’s status, they have a significant role in supporting the parents.^18^ From the physical aspect, stress is a biological agent, a certain amount of which is necessary for the initiation of breast feeding but if its amount is too much for the mother and the baby, it will have opposite results;^9^ stress reduces the secretion of breast milk through reduction of appetite,^19^ the reduction of oxytocin hormone in the mother’s body during feeding^9^ and suppression of prolactin secretion in the mother’s body through adrenergic mechanisms^20^ and if this trend continues it will stop milk’s production.^9^ This causes a disruption in mother-infant attachment together.^21^ Also, reduction in milk production decreases the infant’ weight and the baby is at risk of serious injury, leading to a prolonged NICU hospitalization. Subsequently, the family and the hospital will undergo additional costs. Using milk formula not only deprives the baby from the blessings and benefits of breast milk, but also imposes a high cost on the family. Thus, it exposes the father to more stress and its complications.^22^ Sometimes, these results cause the family to be unwilling to continue treatment; hence, by reducing stress in parents of preterm infants hospitalized in the NICU, we can reduce the complications caused by stress and its direct or indirect sorrow in the infant. So, the awareness of the NICU parent experiences by the medical team is essential to the quality of care^4^ and most of the nurse’s and the medical team’s attention in NICU is focused on the infant. Since to the best of our knowledge no study had been conducted in Fars province, we decided to emphasize another part of the duties of nurses in NICU, i.e. the support of parents, to assess the stress levels of parents of premature infants hospitalized in NICU and to help to reduce it through appropriate training and informing the parents. The level of parental information and understanding varies, so education and presentment of nurses, pattern of information and supportive source are more beneficial than intelligence support.^23^The present study aimed to determine the effect of the educational program on the premature infants’ parental stress in NICU. It was hypothesized that receiving educational program will decrease the parental stress in NICU.

## Materials and Methods

This study was a double-blind randomized controlled trial with a pre-and post-test and follow up design in two intervention and control groups. The setting of the study was NICU in Hazrat Zainab hospitals affiliated to Shiraz University of Medical Sciences (SUMS), Shiraz, Iran. This study was conducted from February 2013 to March 2014. The target population was the parents of premature infants with the age of 28-37 weeks.

The parents of premature infants with the following inclusion criteria participated in the study: (1) age 18 years or above and being literate; (2) a minimum of 1 and maximum of 3 times to meet the baby and visiting the NICU; (3) no previous experience of stressors such as divorce, separation from spouse, incarceration, bereavement, injury or illness, loss of job and family conflicts; (4) no previous experience of a child being hospitalized in the neonatal intensive care unit; (5) according to their own statements not being treated with certain medications such as tranquilizers, and anti-anxiety; (6) according to their own statements, no physical or mental problem inhibiting participation in the study and completion of the questionnaire used in the study; (7) lack of participate in another program or intervention during the study; (8) not being medical staff. Also, they all had single ton infants with the following features: (1) 5-minute Apgar score above 3; (2) infant gestational age of 28-37 weeks; and (3) the infants only with prematurity, apnea, respiratory distress syndrome, or all the three.

Based on the literature review^10^ and effect size=-1.62, power of 0.8, and a=0.05, a 60-parent sample size (30 parents in each group) was determined for this study. A total of 60 parents (n=120) were recruited for the study. Simple randomization procedure was used to allocate the parents into the study groups. In the simple randomization procedure, the samples were randomly assigned to the intervention (30 couples n=60) or the control groups (30 couples n=60) and each participant had the same probability of being assigned to any particular groups.

**Figure 1:**
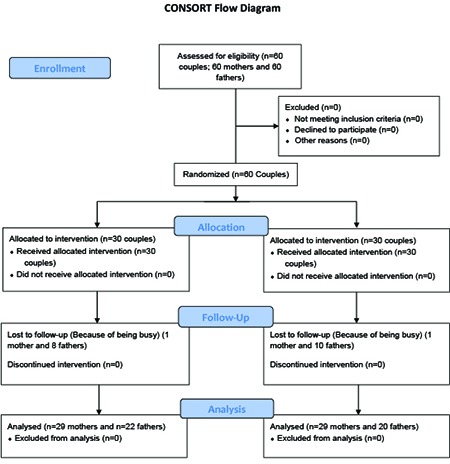
Flow diagram of the participants

During the study period, on the second day after admission, one mother and 8 fathers in the intervention group stopped their participation since they were busy. One mother and 10 fathers in the control group were also excluded for the same reason. Therefore, twenty nine mothers and twenty two fathers in the intervention group participated in the 12 day intervention, while twenty nine mothers and twenty fathers in the control group received the routine care and the study continued with 110 participants (figure 1).

The intervention consisted of four steps as follows:

**First step:** The second day after the admission of the premature infant in the NICU, the researcher visited the parents and explained the objectives of the study for the participants. Then, informed consent, demographic information and a stress questionnaire were completed by parents. This meeting for each parent was separately held in the conference room which was next to the NICU. It should be noted that to avoid stress on the part of the parents, it was arranged that in the case of need, the NICU personnel could call them since they were in the conference room beside the ward.

**Second step**: On the third day after admission, by using Power Point in the form of training slides and booklets, the equipment and unit’s conditions were explained for each parent.

**Third step: **Four days after admission, the participants were provided with information about general condition of the baby and current and future status of the baby by the physician and the researcher in the baby’s bedside.

**Fourth step: **Five days after admission in the room, there was a conference on spouse support education in critical conditions and understanding and knowing each other and working together to solve problems and not blaming each other; then, the parents’ questions were answered. All training sessions were held simultaneously according to a predetermined schedule, face to face for each parent separately about 45-60 minutes based on the parents’ tolerance.

A booklet for parents of premature infants with the content of the topics discussed in the training sessions was prepared, and at the second intervention meeting it was given to them so that they could use it during hospitalization of their baby and different stages of the study.

The parents in the control group completed the scale on the second and fifth days of admission and also seventh days after intervention, as well. During this period, they did not get any training sessions similar to the intervention group and they received the routine treatment and care. At the end of the study, for ethics concern, a similar training booklet was given to each parent participating in the control group. 

The outcome measures in this study consisted of demographic information and parental stress. The parental stress was collected on the second day after admission, the fifth day after admission, and one week after the intervention.

Parental Stressor Scale: Neonatal Intensive Care Unit (PSS: NICU) designed by Mile and edited in 2011, was used to measure the parental stress.^24^ This 26 item scale consisted of three domains: infant behavior and appearance (14 items), sights and sounds (5 items), and parental role alterations (7 items). Itself-report scale scores ranged from 1=not at all stressful, to 5=extremely stressful. Construct validity of the PSS: NICU has been indicated through relationship with measures of state anxiety (r=0.46-0.61, P< 001).^25^

Internal consistency of the PSS: NICU was reported 0.89 to 0.90 for the entire instrument. Busse et al. reported the PSS: NICU domains reliability as sights and sounds=0.74; infant behavior and appearance=0.86; and parental role alterations=0.85.^24^

In the present study, in order to use the English version of PSS: NICU, forward and backward translation was done. At first, an Iranian person translated the scale from English into Persian. Then, the Persian versions were translated back into English by an Iranian person who was living in the United States. After that, the English version was compared with the original version to identify inconsistencies and loss or change of meaning. Then any inconsistencies were resolved.

The content validity of the Persian version of PSS: NICU was approved by 10 nursing and neonatology faculty members in Shiraz University of Medical Sciences. The internal consistency of the Persian version of the PSS: NICU in mothers and fathers was determined by Cronbach’sa of 0.95 and 0.98, respectively. Moreover, in our study, the internal consistency of the PSS: NICU was reported greater than 0.70 for all domains scales (in mothers: sights and sounds=0.88, infant behavior and appearance=0.91, and parental role alterations=0.87; in fathers: sights and sounds=0.85, infant behavior and appearance=0.95, and parental role alterations=0.95).

The study was approved by the Ethics Committee of SUMS, Shiraz, Iran. The aim and the protocol of the study was explained for the parents. The informed consent was obtained from the participants. It was explained that the participation/non-participation of this study were voluntary and did not affect the baby’s treatments and cares.  

In this study, the researcher assistant who collected the data remained blind to the outcome measures and allocation of the parents to the intervention and control groups. Moreover, the statistician who performed the data analysis was masked of the aim of the study and the study groups, as well.

The data were analyzed by using SPSS (version=14). Independent t-test was used to compare the mean of premature infants’ parental stress in the intervention and control groups on the second day after admission, fifth day after the admission, and a week after the intervention. In addition, repeated measures analysis of variance (RM-ANOVA) was used for comparing the premature infants’ parental stress three times (the second day after admission, fifth day after the admission, and a week after the intervention) in two groups. It should be noted that before using independent sample T-Test, their assumptions, such as normality and homogeneity of variance (equal variance), were assessed. Moreover, the two assumptions of RM-ANOVA, i.e. normal distributions of variables, and the same variance for the comparison of any two levels (sphere city) was established in this study. P<0.05 was considered as significant. 

## Results

In both groups, most of the mothers were in 25-29 years of age and fathers were 26-30 years old. As to the jobs, in the two groups, the majority of mothers were housewives and fathers, workers. The common perception of economic status of the parents participating in the intervention group was moderate and in the control it was good.  In both groups, the level of education in most of the mothers and fathers was diploma and college degree. Moreover, in both groups, the type of childbirth in the majority of the subjects was cesarean delivery. As to the infant’s sex, 58.6% males and 41.4% females were in the intervention group and 37.9% males and 62.1% females were in the control group. There were no differences between the intervention and control groups regarding the socio-demographic characteristics (table 1).

**Table 1:** Description of the intervention and control infants and couples (n=30 couples)

** Groups** **Variables**	**Intervention**	**Control**	** c^2^, P value **
	**n (%)**	**n (%)**	
*Mothers’ age * ^††^			
15-20	3 (10.3)	2 (6.9)	0.92, 0.82
21-25	8 (26.7)	9 (31.0)	
26-30	15 (51.7)	13 (44.8)	
≥31	3 (10.3)	5 (17.2)	
*Fathers’ age* ^††^			
21-25	3 (13.6)	0 (0.0)	2.95, 0.22
26-30	10 (45.5)	11 (55.0)	
≥31	9 (40.9)	9 (45.0)	
*Mothers’ Job* ^††^			
Housewive	28 (96.6)	26 (89.7)	1.07, 0.3
Employee	1 (3.4)	3 (10.3)	
*Fathers’ Job* ^††^			
Jobless	1 (4.5)	1 (5.0)	0.12, 0.93
Worker	11 (50.0)	11 (55.0)	
Employee	10 (45.5)	8 (40.0)	
*Parental economic status* ^††^			
Excellent	0 (0.0)	1 (0.5)	2.12, 0.54
Good	6 (27.3)	8 (40.0)	
Moderate	10 (45.5)	7 (35.0)	
Poor	6 (27.3)	4 (20.0)	
*Mothers’ education* ^††^			
High school and lower	8 (26.6)	8 (26.6)	0.0, 1.0
Diploma and College degree	21 (72.0)	21 (72.0)	
*Fathers’ education* ^††^			
High school and lower	7 (31.8)	3 (15.0)	2.54, 0.28
Diploma and College degree	15 (68.3)	17 (85.0)	
*Type of childbirth* ^††^			
Cesarean delivery	22 (75.9)	19 (65.5)	0.74, 0.38
Normal delivery	7 (24.1)	10 (34.5)	
*Infant’s sex* ^††^			
Male	17 (58.6)	11 (37.9)	2.48, 0.11
Female	12 (41.4)	18 (62.1)	

^††^The data of one mother and 8 fathers in the intervention group and one mother and 10 fathers in the control group were missed.

The most common infants’ birth rank was first rank (intervention=%72.4 and control=%65.5); From the birth weight point of view, in the intervention group 44.8% of the infants were reported to have less than 1500 g and 55.2% were greater or equal to 1500 g; in the control group, 44.8%reported to have greater or equal to 1500 g and 55.2% were less than 1500 g. The highest rates of infant age were reported to be between 29-32 weeks of pregnancy in both groups. The mean Apgar scores 5 minutes in the intervention and control groups were 6.86 (SD=1.52) and 6.72 (SD=1.55).No significant dif­ference was shown between the intervention and control groups regarding the mean Apgar score 5 minutes.

In the intervention group, the mean scores of premature infants’ maternal stress on the second day after admission, after the intervention, and a week after the intervention were 94.793±14.283, 77.413±12.899, and 59.724±13.551, respectively. In the control group, the mean scores of premature infants’ maternal stress on the second day after admission, after the intervention and a week after the intervention were 94.482±20.031, 96.793±10.913,and 86.758±12.129,respectively.

**Figure 2:**
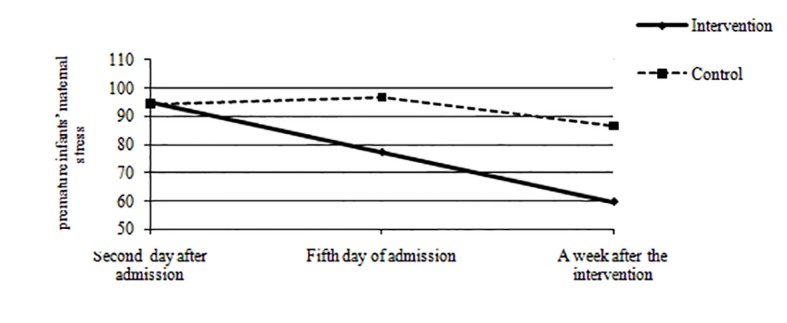
The premature infants’ maternal stress between the intervention and control groups across the three study periods

No significant difference was demonstrated between the two groups regard­ing the mean score of premature infants’ maternal stress on the second day after admission (t=0.06, P=0.94). However, the difference between the two groups on the fifth day of admission, and a week after the intervention of premature infants’ maternal stress was significant (table 2). Moreover, RM-ANOVA indicated a significant difference between the two groups regarding the premature infants’ maternal mean scores of stress during the three study periods (second day after admission, fifth day of admission, and a week after the intervention) in a NICU (F=23.05, P<0.0001), (table 2, figure 2).

**Table 2:** Comparison of the intervention and control groups on Iranian premature infants’ parental stress in the Neonatal Intensive Care Unit

		**Two days** **after admission**	**Five days after** **admission**	**A week after** **the intervention**	**Repeated measure** **Between group; F, P value**
Mothers	Intervention	94.79(14.28)	77.41(12.89)	59.72(13.55)	F=23.05, P<0.0001
	Control	94.48(20.03)	96.79(10.91)	86.75(12.12)	
	t, df, P value	0.06, 56, 0.94	-6.17, 56, <0.0001	-8.00, 56, <0.0001	
Fathers	Intervention	76.77(16.39)	66.13(14.33)	61.22(18.00)	F=17.35, P<0.0001
	Control	92.30(21.95)	92.30(18.01)	84.70(18.46)	
	t, d.f., P value	-2.61, 40, 0.01	-5. 23, 40, <0.0001	-4.16, 40, <0.0001	

**Figure 3:**
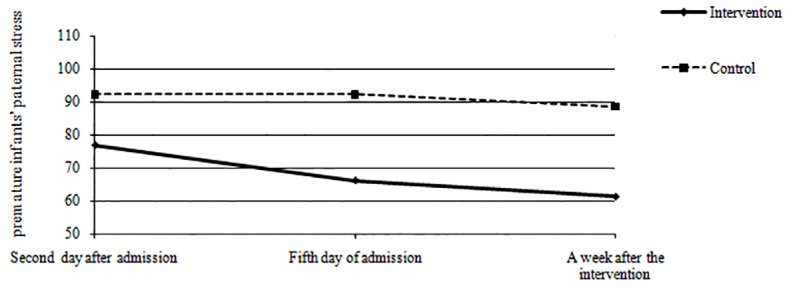
The premature infants’ paternal stress between the intervention and control groups across the three study periods

The mean score of paternal stress on the second day after admission was 76.772±16.390; after the intervention it was 66.136±14.330 and a week after the intervention it was 61.224±18.007. The mean stress score of fathers in the control group on the second day after admission was 92.300±21.957;after the intervention it was 92.300±18.014 and a week after the intervention it was 84.700±18.462 (table 2). RM-ANOVA showed a significant difference between the intervention and control groups regarding the premature infants’ paternal stress during the three study periods (F=17.35, P<0.0001), (figure 3).

## Discussion

This study, with the aim of determining the effect educational program on the parents of premature infants in NICU showed that educational program decreased the mean score of premature infants’ maternal and paternal stress. The results of a study indicated that educational support could reduce the mean stress score of the fathers’ of premature infants hospitalized in Intensive Care Unit while there was no significant change in the mean stress scores of mothers.^26^ Perhaps the reason for the difference between this study and the present study is that the educational support took place a week after admission just in one session while in the present study training sessions were held over two days after admission in the unit during four sessions, and providing information continued till one week after the end of training sessions. The researchers in another study also believed that early intervention is important to reduce stress in parents of preterm infants hospitalized in the NICU.^27^

According to the results of another study also with intervention “Creating opportunities for parent empowerment” revealed that after the first step of intervention, the mean stress scores were reduced in the intervention group, showing a significant difference with the mean stress scores before the intervention. Also, after the second step, mean stress scores decreased in the intervention group and showed a significant difference with the mean stress scores after the first step, but in the control group at this time, there was no significant difference in the mean stress scores. The results of the present study are consistent with that.^23^

To achieve the third objective based on the research findings, the mean score of mothers’ stress in the intervention group a week after the intervention compared with the control group had a significant difference (P=0.001) (59.724 intervention vs. 86.758 control) and the mean stress score of fathers a week after the intervention in the intervention group compared with the control group had also a significant difference (P=0.001) (61.227 intervention vs. 84.7 control).

The intervention of another research conducted as an informational support included a face to face training session about 30 minutes with a visit from the unit by researcher^12^ but answering the following questions was referred to the health care providers. Fathers’ stress score in the intervention group compared with the control group had no significant difference in any of the sub scales. One of the reasons of the differences in their results with the present study might be the training method and its length of time. Lack of sufficient staff leads to an unfavorable condition to respond; as in a qualitative-quantitative research, the fathers considered the health care staff as the main sources of information, but only 50% of them were pleased to receive information from staff.^28^ In the present study, in addition to the oral training sessions and visit of the unit, the researcher was in contact with the parents all throughout the intervention and responded their questions. Therefore, the difference in the results of the present study and those of the mentioned study might be due to the difference in the performance of the intervention.

As a study indicates the parents may not be able to process the information well after preterm birth and they need special care and support, so repetition of the information is very important.^29^ Continuing to provide the necessary explanations and clear answers to parents about the baby’s condition and his status and providing an opportunity to discuss the clinical condition of the new born infant might facilitate a more accurate understanding of the infant and can reduce parental stress levels.^30^

A research citing from another study reports that the amount of information provided for the mothers of premature infants is not clear and this result might also be true about the fathers of premature infants. This study also emphasized the importance of the fathers’ need to get the information.^31^ In addition, they asked for information and its continuous repetition at the right time.

To achieve the fourth objective of the study, the findings showed that the mean score of mothers’ stress in the intervention group on the second day after admission compared with a week after the intervention had a significant difference (P=0.001) and in the mothers in the control group the mean stress score on the second day after admission compared with a week after the intervention was not significant (P=0.12). In the fathers of the intervention group, the mean stress score on the second day after admission compared with a week after the intervention was significant (P=0.001);in the fathers of the control ,the mean stress score was also significant at the same time with the intervention group (P=0.005). Perhaps, reduction in the stress of the fathers of the control group a week after the intervention is due to the influence of passage of the time. However, the reduction of stress, in the fathers of the intervention group is more than those in the control group (76.772 in the intervention group on the second day vs. 61.227 a week later and 92.3 in the control group on the second day vs. 84.7 a week later) and this point may reflect the impact of the continuing education program and the feeling of having a supporter by the parents in the intervention group that causes a significant decrease in parental stress in the intervention group compared with the control group.

Other studies showed that the total parental stress score for mothers in the intervention group compared with the control group was significantly lower, but the fathers’ stress score had no significant difference.^12^^,^^32^ Perhaps, the reasons of the significant difference in the total stress score of fathers in the present study are the 12-day relationship of the researcher with both parents and providing a training handbook for them. In addition, 12 days of continues relationship of the researcher as a supportive nurse with both parents would make information available in the proper time for the parents and repeat it if necessary. Therefore, it is possible that providing a 12-day access to a supportive nurse and also a telephone communication with him can be effective in the results of this study. In addition, as the informal relationship and illegitimate pregnancy are the causes of stress,^33^ perhaps this point accounts for the significant result of parental training intervention in both fathers and mothers in the present study because in the study of Melynke et al. only 53.4% of mothers and 63.3% of fathers of the babies were married and the other parents had no legitimate relation ship.^32^ Because the partners’ strong bonding and commitment toward each other strengthen the family relationships and help them to support each other and manage the crises, only the parents whose child was legitimate participated in the present study.

One of the limitations of this study was the low number of participants in each group therefore, future studies with larger sample sizes are suggested. Another limitation was the fathers’ dropout in the follow up process. 

Other studies should also be conducted on the effect of educational program on development or biological outcome of infants for at least 12 months. Future studies could evaluate the effects of educational program on parental sleep pattern, quality of life, anxiety, depression, and adaptation. 

## Conclusion

This study is important in NICU. It emphasizes the importance of parental education on stress which is common among parents whose infants were admitted in NICU. It highlights the importance of educational intervention in reduction of stress in parents. By using this educational program, parents spend their energy to support and care for their baby instead of dealing with stress. Therefore, it is suggested that healthcare workers arrange such educational program in NICU. 
